# Dietary supplementation of *Aspergillus oryzae* meal and its effect on performance, carcass characteristics, blood variables, and immunity of broiler chickens

**DOI:** 10.1007/s11250-019-01930-1

**Published:** 2019-05-31

**Authors:** Mehdi Zahirian, Alireza Seidavi, Magdalena Solka, Mehran Nosrati, Mirco Corazzin

**Affiliations:** 1 Department of Animal Science, Rasht Branch, Islamic Azad University, Rasht, Gilan 41335-3516 Iran; 2 Department of Genomics, Institute of Genetics and Animal Breeding PAS, ul., Postępu 36A, 05-552 Jastrzębiec, Poland; 3 Department of Agricultural, Food, Environmental and Animal Sciences, University of Udine, Via delle Scienze, 206, 33100 Udine, UD Italy

**Keywords:** *Aspergillus oryzae*, Carcass, Performance, Immune response, Broiler chickens

## Abstract

This study investigated the effect of different levels and consumption periods of *Aspergillus oryzae* meal on performance, carcass characteristics, blood variables, and immunity of broiler chickens. A total of 270 (male and female) Ross 308 chicks were randomly assigned to 9 treatment groups. Two levels (2 g/kg diet and 4 g/kg diet as-fed) of *Aspergillus oryzae* meal (AO) and 4 consumption periods of AO (starter, grower, finisher, and entire period) in a 2 × 4 factorial arrangement were used. Compared with control, AO used during the entire rearing period increased weight gain, reduced relative weight of abdominal fat, aspartate aminotransferase (AST), and alanine aminotransferase (ALT) serum levels, and increased antibody titers against influenza and Newcastle disease vaccination and sheep red blood cells injection. Few differences in the variables considered were found if AO was added to broiler diets only during specific consumption periods, and between the two supplementation levels of AO. In conclusion, the addition of AO to the broiler diet can have beneficial effects in terms of performance, carcass composition, and health, but these positive effects were mainly reached adding AO for the entire rearing period.

## Introduction

All breeding programs for animals, including poultry, are aimed at improving breeding traits, either through genetics (Kawka et al. 2010, 2012) or, for example, nutrition (Nikravesh-Masouleh et al. 2018; Tasirnafas et al. 2015). All of these treatments were aimed at increased body weight gain, growth rate, and conversion efficiency in poultry. Then antibiotics were used in poultry as growth stimulants. The widely use of antibiotics in animal production raised the fears of increasing of the antibiotic resistance of microorganisms. For this reason, the antibiotics used as growth promoters are banned in European Union since 2006 (EC no. 1831/2003). For these reasons, probiotics, prebiotics, and symbiotic are recently used into broiler diets, and there are many studies about their effects on broiler’s performance (Kim et al. 2011). From the fermentation of *Aspergillus oryzae* sp., it is possible to obtain a meal that belongs to the category of prebiotics (Ghiyasi et al. 2007). The use of *Aspergillus oryzae* meal (AO) and oligosaccharides in broiler diets can increase the beneficial microflora, reduce pathogenic bacteria, and increase the digestion of nutrients because of the stimulation of the secretion of enzymes from the stomach and intestinal mucosa. Previous studies do not show clearly the effect of AO on broiler performance. Ghiyasi et al. (2007) showed similar effect of AO to control diet on performance; Amirdahri et al. (2012) have not found a beneficial effect of AO on performance of broilers, while Navidshad et al. (2010) reviewed that AO improves the beneficial microflora and the development of gut.

Moreover, these studies did not concern the effect of AO in specific periods of rearing on performance and health of broiler chickens. Therefore, the objective of this study was to determine the effect of level and consumption period of AO on performance, carcass characteristics, blood variables, and immunity of commercial broilers.

## Material and methods

### Animal, management, and diets

A total of 270 1-day old (male and female) Ross 308 chicks were randomly assigned to 9 treatment groups with 3 replications of 10 animals. Each group was fed for 42 days with iso-caloric and iso-nitrogenous diets (Table 1) that were formulated based on standard recommendation (Ross 2007). Feed were provided ad libitum and in pellet form. Treatments consisted of two levels (2 g/kg diet and 4 g/kg diet as-fed) of commercial AO (Fermacto, PetAg, USA) and four consumption periods of AO (starter, grower, finisher, and entire period). One control treatment without AO was also included.

**Table 1:** Ingredients and chemical composition of used diets

Item	1 to 14 days of age			15 to 28 days of age			29 to 42 days of age		
Experimental group	Control	AO2	AO4	Control	AO2	AO4	Control	AO2	AO4
Ingredients (g/kg as-fed)									
Corn	610	609	617	662	661	660	710	709	708
Soybean meal	320	320	319	287	287	286	236	236	235
Fish meal	29.0	28.7	28.6	10.0	9.8	9.2	10.0	9.7	9.3
Na chloride	2.5	2.5	2.5	3.0	3.0	3.0	3.2	3.2	3.2
Mineral oysters	15.0	15.0	15.0	14.2	14.2	14.2	16.5	16.5	16.5
Ca(22%) P(18%)	13.0	13.0	13.0	13.2	13.2	13.2	13.5	13.5	13.5
Vitamin mineral premix^1^	5.0	5.0	5.0	5.0	5.0	5.0	5.0	5.0	5.0
Methionine	3.4	3.4	3.4	3.5	3.5	3.5	3.5	3.5	3.5
Lysine	2.1	2.1	2.1	2.4	2.4	2.4	2.4	2.4	2.4
Aspergillus meal	0	2	4	0	2	4	0	2	4
Calculated chemical composition (g/kg unless stated otherwise)									
Metabolizable energy (MJ/kg)	12.2	12.2	12.2	12.4	12.4	12.4	12.5	12.5	12.5
Crude protein	223	223	223	200	200	200	180	180	180
Ca (%)	11.1	11.1	11.1	9.4	9.4	9.4	10.0	10.0	10.0
Available P	5.6	5.6	5.6	5.0	5.0	5.0	4.8	4.8	4.8
Lysine (%)	15.0	15.0	15.0	13.0	13.0	13.0	11.0	11.0	11.0
Methionine (%)	7.4	7.4	7.4	6.9	6.9	6.9	6.5	6.5	6.5

^1^One kilogram of premix contained: calcium pantothenate, 4000 mg; niacin, 15,000 mg; vitamin B6, 13,000 mg; Cu, 3000 mg; Zn, 15,000 mg; Mn, 20,000 mg; Fe, 10,000 mg; K, 300 mg; vitamin A, 5 × 10^6^ IU; vitamin D3, 5 × 10^5^ IU; vitamin E, 3000 mg; vitamin K3, 1.5 mg/g; vitamin B2, 1000 mg

Birds received a natural regime for lighting, temperature, and humidity throughout the study period. Ethics approval for the animal trials was obtained from the Animal Ethics Committee, Rasht Branch, Islamic Azad University, Rasht, Iran. Water was provided ad libitum. Animals were vaccinated against bronchitis disease (1 and 7 days of age), Newcastle disease (1 and 7 days of age), influenza disease (1 day of age), and Gumboro disease (21 days of age). For studying the general humoral immune response of animals, sheep red blood cells (SRBC) were injected at 21 days and 35 days of age.

### Sample collection and measurements

Body weight (BW) and feed intake (FI) were measured weekly. Feed conversion ratio (FC) was calculated by dividing feed consumption by body weight gain (WG). In order to assess the humoral immune response to Newcastle and influenza vaccines, one chicken per replication was randomly selected and blood samples were collected at 1, 35, and 42 days of age. Moreover, in order to assess the humoral immune response to SRBC injection, two chickens per replication was randomly selected and blood samples were collected at 28 days and 42 days of age. Blood samples were collected, transferred to the laboratory, and treated as in Davoodi-Omam et al. (2019) and Shabani et al. (2015).

At slaughter, 42 days of age, one representative broiler chicken per replicate was selected and scarified. Breast, drumsticks, spleen, and abdominal fat were removed and weighed; the empty or edible carcass weights were recorded (Shabani et al. 2015). Thighs were also weighed. Relative weights (RW) were calculated as follows: weight of cut or organ (g) / 100 g of body weight.

### Blood and immunity analysis

Glucose, total cholesterol, triglycerides, high-density lipoprotein (HDL), and low-density lipoprotein (LDL) were measured and determined as reported by Shabani et al. (2015). Aspartate aminotransferase (AST, EC 2.6.1.1) and alanine aminotransferase (ALT, EC 2.6.1.2) were assayed according to the method of Reitman and Frankel (1957). The humoral immune response was measured according to the hemagglutination inhibition (HI) method (Seidavi et al. 2014).

### Statistical analysis

Statistical analysis was performed using the general linear models procedure of SPSS for Windows (v 7.5.21). The normality of the data distribution was tested using Shapiro-Wilk test. The model included level and consumption period of AO as main effects and the interaction between main effects. Data were also subjected to one-way ANOVA. Mean separation was accomplished using Duncan post hoc test. All significance level was set at *P* < 0.05.

## Results

The effect of dietary supplementation of AO on feed intake, feed conversation ratio, and weight gain of broiler chickens is showed in Table 2. The lowest feed intake was in the case of broiler chickens fed with the addition of 4 g/kg diet of AO meal. However, in comparison with the control group, not significant differences were found for this variable. Also, no effect of AO level on FC and WG was found. Broiler chickens fed with AO for the entire rearing period showed only numerically lower FC than the other groups, also in comparison with the control group. WG was significantly higher when AO was provided to animals during the entire rearing period than if was provided only during finisher period, and numerically higher than if was provided only during starter or grower periods. The results presented in Table 3 show that the RW of carcass, breast, thighs, spleen, and abdominal fat to the body weight of Ross 308 broilers were not affected by dietary supplementation of AO. However, the diets with AO caused a pronounced decrease in abdominal fat but was highest when the animals were fed with AO only during the starter period.

**Table 2:** Feed intake (FI, expressed as g/d), feed conversion ratio (FC), and weight gain (WG, expressed as g/d) of Ross 308 broilers fed diets with different inclusions of *Aspergillus oryzae* meal (AO), 0 g/kg (control), 2 g/kg (AO2), and 4 g/kg (AO4), from 1 to 14 days of age (starter), from 15 to 28 days of age (grower), from 29 to 42 days of age (finisher), and from 1 to 42 days of age (entire period)

	1 to 14 days of age			15 to 28 days of age			29 to 42 days of age			1 to 42 days of age		
	FI	FC	WG	FI	FC	WG	FI	FC	WG	FI	FC	WG
Control	396.5^a^	1.33^a^	298.1^b^	1222.0^a^	1.67^a^	731.8^b^	2197.5^a^	2.47^a^	889.7^b^	3816.0^a^	1.99^a^	1919.6^b^
AO2-starter	403.5^a^	1.21^a^	333.5^a^	1218.6^a^	1.66^a^	732.2^b^	2193.4^a^	2.46^a^	890.3^b^	3815.6^a^	1.95^a^	1956.0^ab^
AO2-grower	395.0^a^	1.32^a^	299.2^b^	1183.1^a^	1.59^a^	744.2^ab^	2197.9^a^	2.48^a^	886.3^b^	3776.1^a^	1.95^a^	1929.7^b^
AO2-finisher	398.5^a^	1.33^a^	299.6^b^	1226.4^a^	1.68^a^	730.0^b^	2188.2^a^	2.46^a^	889.5^b^	3813.1^a^	1.98^a^	1919.1^c^
AO2-entire period	386.6^a^	1.20^a^	322.2^a^	1198.7^a^	1.58^a^	758.7^a^	2190.3^a^	2.35^a^	932.0^a^	3775.6^a^	1.87^a^	2012.9^a^
AO4-starter	384.4^a^	1.19^a^	322.9^a^	1215.8^a^	1.67^a^	728.0^b^	2195.6^a^	2.48^a^	885.3^b^	3795.8^a^	1.96^a^	1936.2^b^
AO4-grower	392.2^a^	1.32^a^	297.1^b^	1190.8^a^	1.60^a^	747.4^ab^	2192.8^a^	2.44^a^	898.7^b^	3775.8^a^	1.94^a^	1943.2^ab^
AO4-finisher	389.8^a^	1.34^a^	291.0^b^	1208.5^a^	1.66^a^	728.9^b^	2204.6^a^	2.45^a^	899.8^b^	3802.9^a^	1.98^a^	1919.7^c^
AO4-entire period	393.4^a^	1.19^a^	330.6^a^	1192.6^a^	1.60^a^	745.4^ab^	2188.0^a^	2.35^a^	927.1^a^	3774.0^a^	1.88^a^	2003.1^a^
SEM	11.5	0.05	11.5	14.8	0.03	8.9	20.7	0.02	13.8	77.33	0.04	22.2

Means within column and within main effect not sharing a common superscript letter differ significantly (*P* < 0.05)

**Table 3:** Effect of dietary inclusion of *Aspergillus oryzae* meal (AO), 0 g/kg (control), 2 g/kg (AO2), and 4 g/kg (AO4), from 1 to 14 days of age (starter), from 15 to 28 days of age (grower), from 29 to 42 days of age (finisher), and from 1 to 42 days of age (entire period) on carcass and some organs relative to the body weight of Ross 308 broilers

	Eviscerated carcass (%)	Breast (%)	Thighs (%)	Spleen (%)	Abdominal fat (%)
Control	73.0^a^	21.3^a^	23.3^a^	0.19^a^	2.38^a^
AO2-starter	73.8^a^	22.0^a^	22.8^a^	0.20^a^	2.19^a^
AO2-grower	73.3^a^	20.7^a^	23.6^a^	0.21^a^	1.48^b^
AO2-finisher	72.8^a^	21.5^a^	22.4^a^	0.19^a^	1.43^b^
AO2-entire period	74.1^a^	22.2^a^	23.2^a^	0.21^a^	1.52^b^
AO4-starter	72.7^a^	21.7^a^	22.7^a^	0.21^a^	2.15^a^
AO4-grower	73.3^a^	20.8^a^	23.0^a^	0.17^a^	1.48^b^
AO4-finisher	74.6^a^	21.3^a^	23.4^a^	0.18^a^	1.50^b^
AO4-entire period	74.2^a^	22.1^a^	23.2^a^	0.21^a^	1.44^b^
SEM	0.47	0.21	0.15	0.02	0.13

Means within column and within main effect not sharing a common superscript letter differ significantly (*P* < 0.05)

Table 4 summarizes the influence of dietary prebiotic of AO on blood variables such as the following: triglycerides, HDL, LDL and total cholesterol, albumin and total protein, glucose, uric acid, and AST and ALT transferase. Broiler chickens that were fed with AO only during the starter period showed the lowest level of total protein and the highest level of triglycerides and LDL. Feeding animals with AO only during finisher period and during the entire rearing period tended to reduce the total cholesterol level in comparison with the control group and independently by the level of supplementation. In general, in our trial, the use of AO, independently by its level, reduced the RW of abdominal fat, serum cholesterol, triglycerides, and LDL, but not HDL.

**Table 4:** The blood biochemical profiles of Ross 308 broilers fed diets with different inclusions of *Aspergillus oryzae* meal (AO), 0 g/kg (control), 2 g/kg (AO2), and 4 g/kg (AO4), from 1 to 14 days of age (starter), from 15 to 28 days of age (grower), from 29 to 42 days of age (finisher), and from 1 to 42 days of age (entire period)

	Cholesterol (mg/dL)	Triglycerides (mg/dL)	HDL (mg/dL)	LDL (mg/dL)	Total protein (g/dL)	Albumin (g/dL)	Glucose (mg/dL)	Uric acid (mg/dL)	AST^1^ (U/L)	ALT^2^ (U/L)
Control	147.3^a^	76.56^a^	91.42^a^	33.53^a^	4.73^b^	2.37^a^	168.3^a^	3.87^a^	139.0^a^	142.3^a^
AO2-starter	144.5^a^	74.00^a^	87.67^a^	30.25^ab^	4.85^b^	2.42^a^	189.1^a^	3.95^a^	139.0^a^	141.6^a^
AO2-grower	133.4^ab^	56.36^b^	92.23^a^	26.67^b^	5.76^a^	2.56^a^	175.0^a^	3.93^a^	126.8^ab^	136.1^ab^
AO2-finisher	123.7^b^	54.50^b^	85.66^a^	23.74^b^	5.92^a^	2.33^a^	153.3^a^	3.87^a^	128.4^ab^	133.2^ab^
AO2-entire period	121.5^b^	53.65^b^	86.75^a^	24.26^b^	6.00^a^	2.50^a^	186.3^a^	3.82^a^	120.8^b^	123.3^b^
AO4-starter	146.8^a^	74.45^a^	89.17^a^	30.88^ab^	4.90^b^	2.47^a^	186.7^a^	3.78^a^	140.2^a^	139.8^a^
AO4-grower	135.4^ab^	51.76^b^	86.00^a^	24.71^b^	5.83^a^	2.46^a^	156.3^a^	3.75^a^	125.6^ab^	131.5^ab^
AO4-finisher	120.3^b^	52.54^b^	88.45^a^	25.00^b^	5.80^a^	2.52^a^	151.7^a^	3.86^a^	128.2^ab^	135.5^ab^
AO4-entire period	122.3^b^	55.26^b^	85.58^a^	23.26^b^	5.78^a^	2.48^a^	187.3^a^	3.92^a^	119.5^b^	124.5^b^
SEM	4.14	2.98	3.94	1.85	0.23	0.09	8.69	0.05	4.23	3.61

Means within column and within main effect not sharing a common superscript letter differ significantly (*P* < 0.05)

^1^Aspartate amino transferase

^2^Alanine amino transferase

Animals’ immune responses against Newcastle, influenza vaccination, and against SRBC are reported in Table 5. The first two responses were not influenced by the level and period of consumption of AO at 1 day of age. At 35 days of age, animals fed with 4 g/kg diet of AO showed the highest antibody titer. Moreover, in comparison with the control group, AO supplementation tended to increase the antibody titer against both Newcastle and influenza vaccination. At 42 days of age, differences between the experimental groups were not found. AO level did not influence antibody titer both at 28 and 42 days of age (SRBC). However, at 28 days of age, the group fed with 4 g/kg diet of AO for the entire rearing period showed higher value than the control group. At the same age and independently by the supplementation level of AO, the immunoglobulin G (Ig G) were similar between the experimental groups, while broiler chickens fed with AO for the entire rearing period highlighted higher Ig M than the control group. At 42 days of age, animals supplemented with AO only during finisher and the entire rearing period showed higher total immunoglobulin titer than animals fed with AO only during starter or grower period and in comparison with the control group. Animals fed with AO only during starter period reported numerically lower level of Ig G at 28 days of age, while broiler chickens supplemented with AO during the entire rearing period had higher immunoglobulin M (Ig M) at 42 days of age.

**Table 5:** Immune response after vaccination against Newcastle (Nv) and influenza virus (Iv) and after sheep red blood cells (SRBC) in Ross 308 broilers fed diets with different inclusions of *Aspergillus oryzae* meal (AO), 0 g/kg (control), 2 g/kg (AO2), and 4 g/kg (AO4), from 1 to 14 days of age (starter), from 15 to 28 days of age (grower), from 29 to 42 days of age (finisher), and from 1 to 42 days of age (entire period)

	Nv 1 day age	Nv 35 days age	Nv 42 days age	Iv 1 day age	Iv 35 days age	Iv 42 days age	Ig^1^ Total 28 days age	Ig G 28 days age	Ig M 28 days age	Ig Total 42 days age	Ig G 42 days age	Ig M 42 days age
Control	3.66^a^	3.00^c^	4.00^a^	3.00^a^	1.33^c^	1.30^a^	2.09^b^	1.66^a^	1.10^b^	6.35^b^	5.63^a^	0.78^c^
AO2-starter	4.10^a^	3.95^b^	4.23^a^	3.50^a^	1.90^b^	1.78^a^	3.26^ab^	1.46^a^	1.45^ab^	6.78^ab^	5.66^a^	1.75^abc^
AO2-grower	3.96^a^	3.20^ab^	3.33^a^	3.66^a^	2.36^ab^	2.00^a^	3.20^ab^	1.48^a^	1.52^ab^	6.90^ab^	6.00^a^	1.85^ab^
AO2-finisher	4.00^a^	3.66^bc^	3.66^a^	3.70^a^	2.30^ab^	1.70^a^	2.15^b^	1.60^a^	1.00^b^	7.25^a^	6.78^a^	2.15^a^
AO2-entire period	4.10^a^	4.56^a^	4.45^a^	3.50^a^	3.60^a^	1.56^a^	3.35^ab^	1.50^a^	1.80^a^	7.30^a^	6.46^a^	2.50^a^
AO4-starter	4.00^a^	3.90^b^	4.23^a^	3.64^a^	2.35^ab^	1.80^a^	3.31^ab^	1.38^a^	1.48^b^	6.85^ab^	5.50^a^	1.55^abc^
AO4-grower	3.90^a^	4.13^ab^	3.43^a^	3.65^a^	3.60^a^	1.50^a^	3.27^b^	1.53^a^	1.56^ab^	6.80^ab^	5.95^a^	1.83^ab^
AO4-finisher	4.00^a^	3.70^bc^	3.50^a^	3.40^a^	2.60^ab^	1.80^a^	2.13^b^	1.47^a^	1.05^b^	7.42^a^	6.32^a^	2.10^a^
AO4-entire period	4.00^a^	4.45^a^	4.56^a^	3.70^a^	3.66^a^	2.00^a^	3.38^a^	1.63^a^	1.76^a^	7.46^a^	6.67^a^	2.45^a^
SEM	0.50	0.83	0.43	0.40	0.35	0.40	0.28	0.16	0.22	0.55	0.42	0.29

Means within column and within main effect not sharing a common superscript letter differ significantly (*P* < 0.05)

^1^Immunoglobulin

## Discussion

Several studies have shown the beneficial effects of *Aspergillus*-originated prebiotics on poultry performance. AO added to broiler diets for the entire rearing period allowed obtain animals with higher WG and numerically lower FI. This result may indicate that the main effect of AO, from the performance point of view, is to increase the digestion of nutrients. Our results are in agreement with the findings of Falaki et al. (2011) that, considering a supplement level of 2 g/kg diet of AO, observed a significant increase of the body weight of broiler chickens. From 1 to 14 days of age, the animals fed with AO showed WG higher than the other groups. Moreover, from 1 to 42 days of age and in comparison with the control group, the greatest WG were obtained from broiler chickens that were fed with AO during the entire rearing period regardless of the level used. Navidshad et al. (2010) highlighted an increase of body weight of broiler chickens if AO was included in the diets at 3 g/kg, but not at 1.5 g/kg level. However, in our trial, a supplementation level of 2 g/kg diet seems sufficient in order to improve the WG of broiler chickens. No significant effect of AO level on FC was found.

In turn, the RW of spleen was similar between the experimental groups. Amirdahri et al. (2012) stated that AO affects abdominal reduction because it favors the growth of *Bacillus subtilis*. Moreover, Amirdahri et al. (2012) explained that mannanoligosaccharides contained in AO favor the growth of lactic acid–producing bacteria that increase the deconjugation of bile acids. Also, Navidshad et al. (2010) showed a higher RW of abdominal fat of broiler chickens fed with 3 g/kg diet of AO.

In comparison with the control group, the serum total protein was increased by the use of AO independently by the level, with the exception of the use of AO only during the starter period. On the basis of the available literature, this result is not easy to explain. Indeed, considering mannanoligosaccharides supplementation, Houshmand et al. (2011) showed a positive effect on protein digestibility; conversely, Shafey et al. (2001) have not found any effect. When AO was added to the diets for the entire rearing period, broiler chickens showed the lowest level of AST and ALT. AST and ALT are enzymes, and their presence in the blood can indicate a hepatic damage or injury (Abd 2014). Consequently, the use of AO in the diets of broiler chickens can have a beneficial effect reducing the liver stress. Not in agreement with our results, Yalçinkaya et al. (2012) and Yalçin et al. (2014) showed that serum AST and ALT were not affected by dietary supplementation of diets with mannanoligosaccharides that derived from the cell wall of yeast, and by dietary supplementation of yeast cell wall respectively. Houshmand et al. (2012) found that mannanoligosaccharides were not able to influence the antibody response to Newcastle disease vaccination; moreover, Shahir et al. (2014) have not found an effect of mannanoligosaccharides also on the antibody response to influenza vaccination. Sugiharto (2014) reviewed that prebiotics can enhance the immune response of chicken, but the mechanism of improving of the immune response by AO is not clear.

## Conclusion

The addition of AO to the broiler chickens’ diet at a level up to 4 g/kg can have some beneficial effects in terms of in vivo performance, carcass composition, and animals’ health. Indeed, AO offered for the entire experimental period improved the WG, reduced the abdominal fat, and reduced serum AST and ALT. Moreover, AO can have a beneficial effect on broiler chickens’ immunity especially if added at a level of 4 g/kg diet for the entire experimental period.
